# Tetranuclear Oxo-Titanium Clusters with Different Carboxylate Aromatic Ligands: Optical Properties, DFT Calculations, and Photoactivity

**DOI:** 10.3390/ma11091661

**Published:** 2018-09-08

**Authors:** Maciej Janek, Aleksandra Radtke, Tadeusz M. Muzioł, Maria Jerzykiewicz, Piotr Piszczek

**Affiliations:** 1 Faculty of Chemistry, Nicolaus Copernicus University in Toruń, ul. Gagarina 7, 87-100 Toruń, Poland; maciejjanekk@gmail.com (M.J.); Aleksandra.Radtke@umk.pl (A.R.); tadeuszmuziol@wp.pl (T.M.M.); 2 Nano-implant Ltd. Gagarina 5/102, 87-100 Toruń, Poland, NIP 9562314777; 3 Faculty of Chemistry, Wrocław University, ul. F. Joliot-Curie 14, 50-383 Wrocław, Poland; maria.jerzykiewicz@chem.uni.wroc.pl

**Keywords:** titanium(IV) oxo-clusters, photoactivity, band gap modification, photoluminescence, DFT calculations, composite materials

## Abstract

Titanium(IV) oxo-clusters of the general formula (Ti_4_O_2_(O^i^Bu)_10_(O_2_CR’)_2_) (R’ = C_13_H_9_ (**1**), PhCl (**2**), PhNO_2_ (**3**)) were studied in order to estimate their potential photoactivity. The structure of the resulting tetranuclear Ti(IV) oxo-complexes was then determined via single crystal X-ray diffraction, infrared and Raman spectroscopy, and electron spin resonance (ESR). An analysis of diffuse reflectance spectra (DRS) allowed for the assessment of band gap values of (**1**)–(**3**) microcrystalline samples complexes. The use of different carboxylate ligands allowed the band gap of tetranuclear Ti(IV) oxo-clusters to be modulated in the range of 3.6 eV–2.5 eV. Density functional theory (DFT) methods were used to explain the influence of substitutes on band gap and optical activity. Dispersion of (**1**)–(**3**) microcrystals in the poly(methyl methacrylate) (PMMA) matrixes enabled the formation of composite materials for which the potential photocatalytic activity was estimated through the study on methylene blue (MB) photodegradation processes in the presence of UV light. The results obtained revealed a significant influence of carboxylate ligands functionalization on the photoactivity of synthesized tetranuclear Ti(IV) oxo-complexes.

## 1. Introduction

The unique physicochemical and biological properties of titanium dioxide favor its wide application in a variety of fields in our lives. The optical properties and photocatalytic activity of materials based of TiO_2_ have especially been intensively studied in recent times [1,2,3]. Titania photoactivity is utilized in water splitting, purification of air and water, reduction of environmental pollutants, and in antimicrobial applications [4,5]. Recently, much attention has been devoted to the use of titanium oxo-clusters (TOCs) as compounds exhibiting similar properties to TiO_2_ but characterized by a discrete molecular structure [6,7,8,9,10]. An analysis of the literature indicates the significance of TOCs in synthesis of inorganic–organic hybrid materials that are produced through introduction of metal oxo-clusters into the polymer matrix [11]. The possible interactions between inorganic and organic components may result in an improvement of structural properties of the polymer as well as its thermal, mechanical, and barrier properties due to cross-linking and filling. The unique properties of oxo-clusters, e.g., photochromicity, catalytic/biological or magnetic activity, can give entirely new properties to the composite material compared to the base polymer [11,12,13,14,15,16,17,18,19,20,21,22]. Therefore, studies on TOC synthesis of the titanium oxide core with the desirable architecture, size, and physicochemical properties are important for the production of novel inorganic–organic composite materials [17].

A good example of the above is the studies on TOCs, which are stabilized by phosphonate and different carboxylic acids used as photocatalysts in the process of water splitting and photodegradation of organic dyes [22]. According to these investigations, the structural functionalization of carboxylate ligands associated with the size modification of the band gap had a direct impact on the photocatalytic activity of oxo-clusters. In addition, the use of (Ti_6_O_6_(O^i^Pr)_6_(O_2_CR’)_6_) (R’ = C_6_H_4_NH_2_, C_6_H_4_NHMe, C_6_H_4_NMe_2_, C_6_H_3_(F)NH_2_, C_6_H_3_(Cl)NH_2_) complexes in the photocatalytical degradation of methylene blue (MB) and rhodamine B (RB) revealed a clear influence of the functionalization method of carboxyl groups on the energy band gap size and photocatalytic activity of these compounds [23]. Lin et al. described three TOCs with {Ti_6_O_4_} core consisting of pivalic and benzoate ligands as photocatalysts bearing better water splitting properties than pure TiO_2_ nanowires, nanotubes, and nanosheets phases [24]. Application of phthalic acid in synthesis of TOCs led to the formation of the oxo-complex, which contain {Ti_6_O_3_} cores, which was used in the degradation of methyl orange in the presence of H_2_O_2_ [25]. In addition, {Ti_6_O_3_} clusters with malonate and succinate ligands were studied for photodegradation of methyl orange [26].

In our previous works, we have focused on synthesis, structure determination, and photocatalytic activity studies of tetranuclear oxo-complexes [19,27]. The results of earlier studies revealed that three different types of (Ti_4_O_b_(OR)_c_(O_2_CR’)_16−2b−c_) complexes are usually formed in a direct reaction of Ti(OR)_4_ and organic acid in the different alkoxide/acid molar ratio, i.e., (a) {Ti_4_O_4_} of C.r. = 1 (C.r.—complexation ratio, e.g., (Ti_4_O_4_(OR)_4_(O_2_CR’)_4_), R = ^i^Pr, ^t^Bu, R’ = H, ^i^Pr, C(Me)_2_Et, Co_3_C(CO)_9_ [19,21]); (b) {Ti_4_O_2_} of C.r. = 1.5 (e.g., (Ti_4_O_2_(OR)_6_(O_2_CR’)_6_), R = ^i^Pr, R’ = C_2_H_3_, C(Me) = CH_2_, C_5_H_4_FeCp [21]); and (c) {Ti_4_O_2_} of C.r. = 0.5 (e.g., (Ti_4_O_2_(OR)_10_(O_2_CR’)_2_), R = ^i^Pr, ^i^Bu, R’ = H, C_6_H_4_NH_2_ [21,20,27]). Moreover, Liu et al. proved the formation of (Ti_4_O_3_(O^i^Pr)_6_(fdc)_2_) complex, which consists of square-planar {Ti_4_(µ_4_-O)(µ-O)_2_} cores (C.r. = 0.75) in the hydrothermal conditions (the reaction of Ti(O^i^Pr)_4_ with 1,1-ferrocenedicarboxylate acid (fdcH_2_) in DMF) [28]. The structures of Ti(IV) oxo-complexes containing {Ti_4_O)} cores (C.r. = 0.25) were also found, e.g., (Ti_4_O(OEt)_12_(R’)) (C.r. = 0.25, R’ = O_3_P-Phen, ^i^BuPO_3_), (Ti_4_(µ_3_-O)(O^i^Pr)_8_(O_3_P-^t^Bu)_3_(DMSO)) [20].

From the abovementioned tetranuclear systems, the (Ti_4_O_2_(O^i^Bu)_10_(OOCC_6_H_4_NH_2_)_2_) was especially interesting for us. This complex was synthesized with a good yield in a direct reaction of Ti(O^i^Bu)_4_ with 4-aminobenzoic acid in molar ratio 4:1 at room temperature [27]. The isolated crystals revealed hydrophobic properties and significantly lower sensitivity to moisture in comparison to compounds such as (Ti_4_O_4_(OR)_4_(O_2_CC(Me)_2_Et)_4_) or (Ti_6_O_6_(OR)_6_(O_2_CC(Me)_2_Et)_6_) (R = ^i^Bu, ^t^Bu) [19]. Moreover, the (Ti_4_O_2_(O^i^Bu)_10_(O_2_CC_6_H_4_NH_2_)_2_) compound revealed a promising activity in photocatalytic MB degradation under UV light irradiation. Having considered previous reports on the influence of the type of carboxylate ligand on the optical properties and photocatalytic activity of oxo-clusters, we decided on the synthesis of new (Ti_4_O_2_(O^i^Bu)_10_(O_2_CR’)_2_) complexes with different carboxylate ligands, i.e., fluorene-9-carboxylate (O_2_CC_13_H_9_), 3-chlorobenzoic carboxylate (O_2_CC_6_H_4_Cl), and 3-nitrobenzoic carboxylate (O_2_CC_6_H_4_NO_2_). Besides synthesis of new tetranuclear Ti(IV) oxo-complexes and their structural and spectral characteristic, it was interesting to determine the impact of carboxylate ligands on the {Ti_4_O_2_} core structure, band gap, optic properties, and photocatalytic activity.

## 2. Materials and Methods

### 2.1. General Information

Titanium(IV) isobutoxide (Aldrich, St. Louis, MO, USA), 3-chlorobenzoic acid (Aldrich), 3-nitrobenzoic acid (Aldrich), 4-aminobenzoic acid (Aldrich), and fluorine-9-coarboxylic acid (Organic Acros, Geel, Belgium) were purchased commercially and were used without purification. The solvents applied—toluene, tetrahydrofuran, and acetone—were distilled before their use. All operations were performed in an argon atmosphere using standard Schlenk techniques. The vibrational spectra of synthesized compounds were registered using the Perkin Elmer Spectrum 2000 FT-IR spectrometer (400–4000 cm^−1^ range, KBr pellets) and the RamanMicro 200 spectrometer (PerkinElmer, Waltham, MA, USA). ^13^C NMR spectra were carried out on Bruker Advance 700 (Madison, WI, USA). Elemental analyses were performed on Elemental Analyzer vario Macro CHN Elementar Analysensysteme GmbH (Langenselbold, Germany). The solid state optical diffuse reflection experiment was carried out on the Jasco V-750 Spectrophotometer (JASCO Deutschland GmbH, Pfungstadt, Germany) equipped with an integrating sphere for diffuse reflectance spectroscopy. The recorded spectra were analyzed using Spectra Manager^TM^ CFR software (JASCO Deutschland GmbH, Pfungstadt, Germany). The solid-state luminescence spectra were recorded on Gilden λ Photonics (Glasgow, UK). All ESR spectra were recorded at room temperature using a Bruker Elexsys E500 spectrometer (Rheinstetten, Germany) at a microwave power of 20 mW and modulation amplitude of 0.5 G. In the case of low intensity signals, five scans were accumulated. The measurements were performed for powdered samples immobilized on poly(methyl methacrylate) (PMMA) foils. Samples on foils were studied using special EPR Tissue Sample Cells (Wilmad-Labglass, WG-807 type, Vineland, NJ, USA). All samples were irradiated in situ using the 100 W mercury lamp (Bruker ER 203UV Irradiation System, Rheinstetten, Germany). To determine the diagonal components of g-tensor programs, SimFonia 1.26 developed by Bruker was employed.

### 2.2. Syntheses

The synthesis of (Ti_4_O_2_(O^i^Bu)_10_(O_2_CC_13_H_9_)_2_) (**1**): 0.184 g of 9-fluorenecarboxylic acid (0.875 mmol) was added to the solution of 1.19 g titanium(IV) isobutoxide (3.5 mmol) in 2 mL of acetone. Reactants underwent rapid reaction, leading to clear brownish solution. The solution was left for crystallization. Slow evaporation under an inert gas atmosphere led to crystals suitable for X-ray diffraction experiment. The yield basing on acid: 77% (0.46 g). Anal. Calcd. for C_68_H_108_O_16_Ti_4_: C, 59.28; H, 7.93; Ti, 13.94. Found: C, 58.92; H, 7.81; Ti, 13.79.

The synthesis of (Ti_4_O_2_(O^i^Bu)_10_(O_2_CC_6_H_4_Cl)_2_) (**2**): 0.137 g of 3-chlorobenzoic acid (0.875 mmol) was added to the solution of 1.19 g titanium(IV) isobutoxide (3.5 mmol) in 2 mL of toluene, leading to a colorless solution. The solution was left for crystallization. Slow evaporation under an inert gas atmosphere led to crystals suitable for X-ray diffraction experiment. The yield basing on acid: 88% (0.49 g). Anal. Calcd. for C_54_H_98_O_16_Cl_2_Ti_4_: C, 51.24; H, 7.80; Ti, 15.13. Found: C, 50.89; H, 7.98; Ti, 14.95.

The synthesis of (Ti_4_O_2_(O^i^Bu)_10_(O_2_CC_6_H_4_NO_2_)_2_) (**3**): 0.146 g of 3-nitrobenzoic acid (0.875 mmol) was added to the solution of 1.19 g titanium(IV) isobutoxide (3.5 mmol) in 2 mL of THF, leading to a weak yellow solution. The solution was left for crystallization. Slow evaporation under an inert gas atmosphere led to crystals suitable for X-ray diffraction experiment. The yield basing on acid: 94% (0.53 g). Anal. Calcd. for C_54_H_98_O_20_N_2_Ti_4_: C, 50.40; H, 7.68; N, 2.18; Ti, 14.88. Found: 50.94; H, 7.74; N, 2.22; Ti, 14.71.

The synthesis of (Ti_4_O_2_(O^i^Bu)_10_(O_2_CC_6_H_4_NH_2_)_2_) (**4**): Complex of a given formula was synthesized, as reported [27].

### 2.3. X-ray Crystalography Study

For single crystals, the diffraction data of (**1**) and (**2**) were collected using BL14.3 beamline (Helmholtz Zentrum Berlin, Germany, Bessy II), radiation λ = 0.89429 Å, at liquid nitrogen temperature, whereas for (**3**), the diffraction experiment was performed at room temperature using Oxford Sapphire CCD diffractometer, MoKα radiation λ = 0.71073 Å. The data were processed using CrysAlis [29], *xdsapp* [30], and XDS [31], and the numerical absorption correction was applied for all crystals. The structures of all complexes were solved by direct methods and refined with full-matrix least-squares procedure on F^2^ (SHELX-97 [32]). All heavy atoms were refined with anisotropic displacement parameters. The positions of hydrogen atoms were assigned at calculated positions with thermal displacement parameters fixed to a value of 20% or 50% higher than those of the corresponding carbon atoms. For (**2**) and (**3**), some constraints (DFIX, DANG and ISOR) were applied for C–Cl, C–O, and C–C bonds. In (**1**) the alternate positions were found only for one –O^i^Bu (O61), whereas for (**2**) a positional disorder was detected for chlorine atoms in O201, O211, and O221 carboxylate anions and in O81, O131 and O171 –O^i^Bu ligands. In (**3**), we observed a significant disorder of O71, O91, O101, O121, O131, O141 and O191 –O^i^Bu. In the final model of (**3**), O141 isobutanolate was incomplete—there was lack of carbon and hydrogen atoms from minor orientation. All figures were prepared in DIAMOND [33] and ORTEP-3 [34]. The results of the data collections and refinement are summarized in Table 1. The crystallographic data of the complex (**4**) were presented in our earlier paper [27].

### 2.4. Preparation and Photoactivity Study of Composites

Hybrid PMMA + {TOCs} composite foils containing 20 wt % of synthesized oxo-clusters were prepared by dissolving 1.0 g of polymer in 5 mL of THF and adding a solution of 0.25 g of TOCs—(**1**), (**2**), (**3**), and (**4**)—in 1 cm^3^ of THF to this solution. The resulting mixtures were stirred, poured into a glass Petri dish and left for evaporation of the solvent. Then, the prepared polymer foils were characterized by Raman and NMR spectroscopy and scanning electron microscopy.

The photoactivity properties of polymer composite foils were studied through MB degradation. Foil samples of size 8 mm × 8 mm were placed in quartz cuvettes, and MB solution (V = 3 cm^3^, initial concentration C_0_ = 1.0 × 10^−5^ M) was poured on films. The prepared samples were exposed to UV irradiation (18 W, range of 340 nm–410 nm with maximum at 365 nm). MB absorbance at 660 nm was registered after 0, 24, 48, 72, 94, 168, and 192 h of irradiation. For the purpose of determination of photoactivity, the methodology of chemical kinetics assuming a Langmuir–Hinshelwood (L–H) reaction mechanism was used. However, the linear dependencies of *A* = *f*(*t*) suggested that *Kc* << 1 in the L–H equation and it could be simplified. Thus, the rate of reaction can be expressed as:

*r* = −*dc*/*dt* = *k*_deg_*Kc*/(1 + *Kc*) ≈ *k*_deg_*Kc* = *k*_obs_*c* (1)

where *c* is a reactant (MB) concentration, *t* is time of the concentration measurement, *k*_deg_ is the rate constant of MB degradation on the surface, *k*_obs_ is a first order observed rate constant, and *K* describes the reactant adsorption process.

### 2.5. The Computational Details

The density functional theory (DFT) calculations were performed with Gaussian09 packages [35]. The HSE06 functional was used with 6-31G(d,p) basis set. The crystal structures were used as an optimization starting geometry. In order to reduce the complexity of calculations, isobutyl moieties were substituted with methyl groups. The criterion of no imaginary frequencies was used to confirm the result as an actual minimum. Density-of states (DOS) plots were visualized with GaussSum 3.0 [36].

## 3. Results

### 3.1. Structures of (Ti_4_O_2_(O^i^Bu)_10_(O_2_CR’)_2_) Clusters

**Figure 1:**
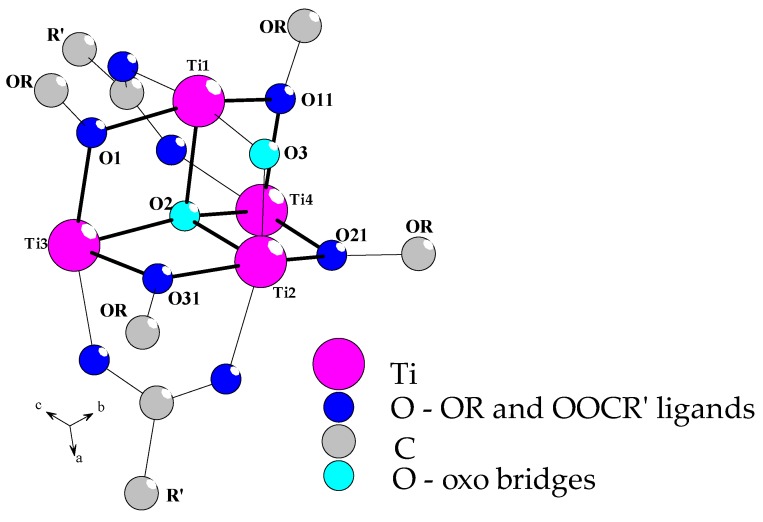
The structure of {Ti_4_O_2_} core, which was found in (Ti_4_O_2_(OR)_10_(O_2_CR’)_2_) (R = ^i^Bu, R’ = C_13_H_9_ (**1**), PhCl (**2**), PhNO_2_ (**3**)) complexes (crystallographic ball-stick scheme). For clarity, the terminal alkoxide groups are omitted.

Structural studies of (**1**)–(**3**) complexes proved that their central part was formed by {Ti_4_O_2_} core that consisted of two µ-oxo bridges, i.e., µ_4_–O connecting all four Ti(IV) atoms in distorted tetrahedral environment and µ–O bridging two of the closest titanium atoms (Figure 1) similar to the structure of (**4**) [27].

Simultaneously, the {Ti_4_O_2_} skeleton was stabilized by four µ_2_-O^i^Bu bridges and two *syn–syn* carboxylate ligands (–O_2_CR’, R’ = C_13_H_9_ (**1**), PhCl (**2**), PhNO_2_ (**3**)). The selected bond lengths and angles, which were found in structures of (**1**)–(**3**) compounds, are listed in Table 2. Comparison of the structural data of these complexes and (**4**) [27] made it possible to trace the influence of carboxylate groups on the geometry of the {Ti_4_O_2_} core, which may have a direct impact on their photocatalytic activity. According to earlier reports concerning photoactivity of Ti(IV) oxo-clusters, the possible changes of {Ti_2_(µ_2_–O)} bridges angle, which can be responsible for the facilitation of the photocatalytic process, should be noted [26]. In the group of synthesized compounds, the values of these angles changed in the row: 105.7(2) (**4**) [27] < 106.09(15) (**1**) < 106.5(2) (**2**) < 107.36(15) (**3**) (Table 2). It suggests that photoactivity of oxo-clusters should also change according to the above dependence. Changes of {Ti_2_(µ_2_–O)} bridges angle were associated with the differences in Ti1–Ti2 distances ((**1**) < (**2**) < (**3**) < (**4**) (2.9521(13) [27]). At the same time, the geometry of {Ti_2_(µ_4_–O)} bridges also changed, which was reflected by differences in Ti3–Ti4 distances and Ti1–O2–Ti2 and Ti3–O2–Ti4 angles (Table 2).

### 3.2. Analysis of Vibrational Spectra

The possible influence of the carboxylic groups on the structure of (Ti_4_O_2_(O^i^Bu)_10_(O_2_CR’)_2_) clusters were studied using IR and Raman spectroscopy. The position of the bands derived from the vibrations of carboxylate ligands (ν_as_(COO) and ν_s_(COO) at ~1600 and ~1400 cm^−1^) and {Ti_4_O_2_} cores (specific bridges: Ti(µ–O), Ti(µ_4_–O) at 400–700 cm^−1^) were especially important for us (Table 3).

Earlier studies of coordination compounds with {Ti_4_O_2_} moiety revealed that the M–O stretches region of this type of compound is represented by many broad bands derived of different M–O modes between 400 cm^−1^ and 800 cm^−1^ [27,37]. The same pattern is shown for compounds bearing {Ti_4_(µ_4_–O)} core [38,39]. Our earlier investigations involving comparison of experimental spectra and DFT calculations of (Ti_4_O_2_(O^i^Bu)_10_(ABZ)_2_) cluster (**4**) have revealed that asymmetric and symmetric Ti(µ–O) bridge bands are located between 700 cm^−1^ and 680 cm^−1^ and Ti(µ_4_–O) in ~630 cm^−1^ and ~535 cm^−1^ [27]. Bands in 700–690 cm^−1^ come from symmetric ν_s_(Ti–O–Ti) modes; ones at ~630 cm^−1^, ~535 cm^−1^ and 430–420 cm^−1^, which were found in IR and Raman spectra of this compound, can be assigned as well as ν_s_(Ti–O–Ti) modes. DFT calculations (HSE06/6-31G(d,p) level of theory) were also carried out for the studied systems consisting of {Ti_4_O_2_} core linked with two carboxylate ligands—(**1**)–(**3**)—and stabilized by ten alkoxide groups (experimental and calculated structural data of {Ti_4_O_2_} cores are compared in Table S1). Results of these studies are summarized in Table 4. According to these data, the frequency of symmetric (s) and asymmetric (a) stretching modes of Ti–O–Ti bridges should be 699–706 cm^−1^ for µ–O bridges and 439–622 cm^−1^ for µ_4_–O ones. The bands, which can be attributed to abovementioned modes, were also found in IR and Raman spectra of (**1**)–(**3**) complexes (Table 3, Figure S1). Analysis of spectral data exhibited that a significant effect of carboxylic groups on the vibration frequency of Ti–O–Ti bridges was not observed.

### 3.3. UV-Vis Absorption Spectra, Band Gap Determination and DOS

**Figure 2:**
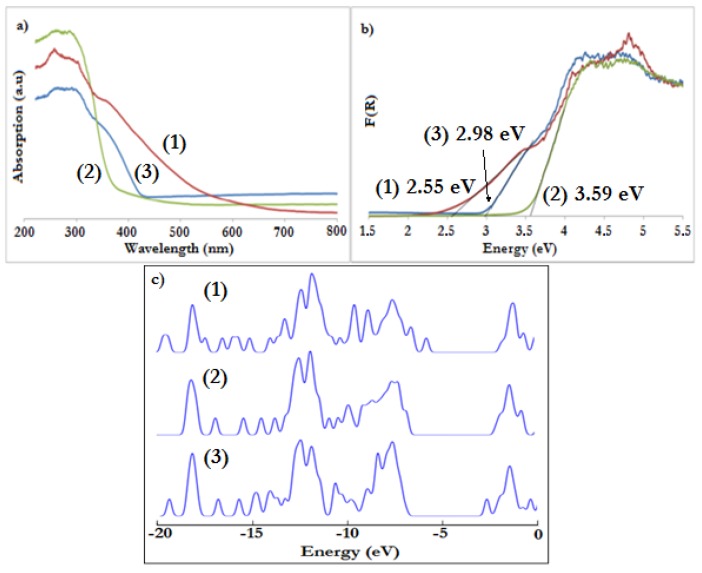
(**a**) Solid-state UV-Vis absorption spectra of (**1**)–(**3**) micrograins; (**b**) Kubelka–Munk function versus light energy plot used for band gap determination; (**c**) density-of states (DOS) plots calculated with HSE06/6-31G(d,p) level of theory for (Ti_4_O_2_(OMe)_10_(OOC-C_13_H_9_)_2_) (**1**), (Ti_4_O_2_(OMe)_10_(OOC-C_6_H_4_-Cl)_2_) (**2**), and (Ti_4_O_2_(OMe)_10_(OOC-C_6_H_4_-NO_2_)_2_) (**3**).

The solid state UV-Vis absorption spectra of studied compounds were recorded with an integrating sphere fitted spectrophotometer at room temperature (Figure 2a). MgO was used as a standard reference. Figure 2b shows the plot of Kubelka–Munk (K–M) function versus light energy, i.e., K = f(hν) where K = (1 − R)^2^/2R and R is reflectance that was used for the optical band gap determination [40,41].

Compound (**3**) absorbed exclusively in UV region of the spectrum, which corresponded to band gap of 3.59 eV. According to theoretical calculations, the chloro-functionalized derivative (**2**) introduced additional band as HOMO, but it didn’t change the value of resulting band gap by a big margin (Figure 2c, Figure S2). By contrast, DOS plots of nitro- and fluorene-derivative showed deep penetration of the gap by functionalities, and reduction of the band gap was therefore evidenced. It is noteworthy that 3-nitrobenzoic derivative (**3**) introduced new states as LUMO, while compound with fluorene (**1**) changed HOMO of the corresponding compound (Figure S3).

Theoretically calculated band gaps were qualitatively reflected in absorption spectra of (**1**) and (**3**), where the bands penetrated into visible region of spectrum (Table 5). The band gaps of (**1**) and (**3**) were estimated by K–M function onset to be 2.98 eV and 2.55 eV, respectively. Calculated and experimentally determined values of band gaps are presented in Table 5. There was a clear overestimation of the energy gap between the values determined experimentally and theoretically; however, this agrees with the tendency of hybrid density functionals to overestimate predicted band gaps [42]. Additionally, the comparison of experimental vs. theoretical values showed that they follow a linear trend (R^2^ = 0.9968), indicating correlation between them. The band gap of (**4**) was determined in previous research to be 2.57 eV [27].

### 3.4. Photoluminescent Properties

**Figure 3:**
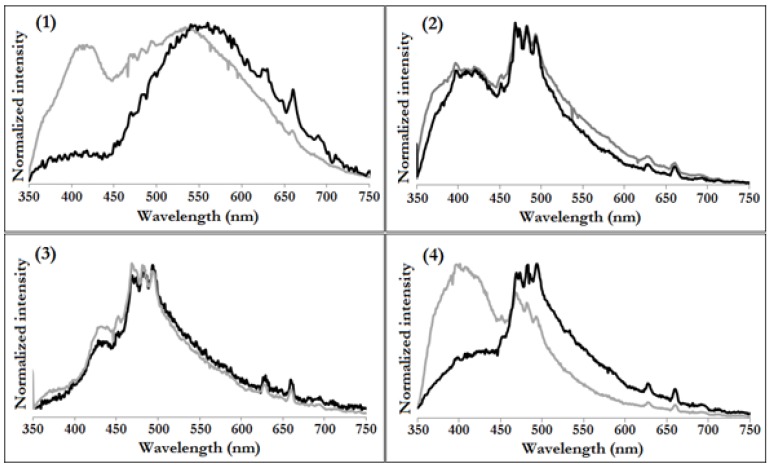
Solid state photoluminescence spectra (1–4) of (Ti_4_O_2_(O^i^Bu)_10_(O_2_CR’)_2_) (R’ = C_13_H_9_ (**1**), PhCl (**2**), PhNO_2_ (**3**), PhNH_2_ (**4**)) respectively (black plots) collected by 330 nm excitation at room temperature compared to spectra of corresponding acids (grey plots).

Solid-state photoluminescence spectra of (**1**)–(**4**) were recorded with samples excited by wavelength of 330 nm (3.76 eV), i.e., energy higher than determined for band gaps (Figure 3). Resulting spectra were compared with the data of corresponding acids. Results showed how coordination to oxo-titanium cluster influenced ligands PL spectra. In the case of (**1**) and (**2**), the coordination to oxo-titanium core causes the decay of higher energy part of the corresponding acid spectrum, indicating interactions of the frontier orbitals of carboxylate with oxo-titanium core. The change in band location was seen in case of fluorene derivative (**2**) where the emission of fluorine-9-carboxylic acid at 540 nm shifted toward 560 nm when coordinated. This type of conversion was not witnessed for other ligands. Interestingly, photoluminescent spectra of (**3**) and (**4**) didn’t show any distinct changes when compared to spectra of acids.

### 3.5. Preparation and Characteristic of Polymer Composites (PMMA + TOCs)

**Figure 4:**
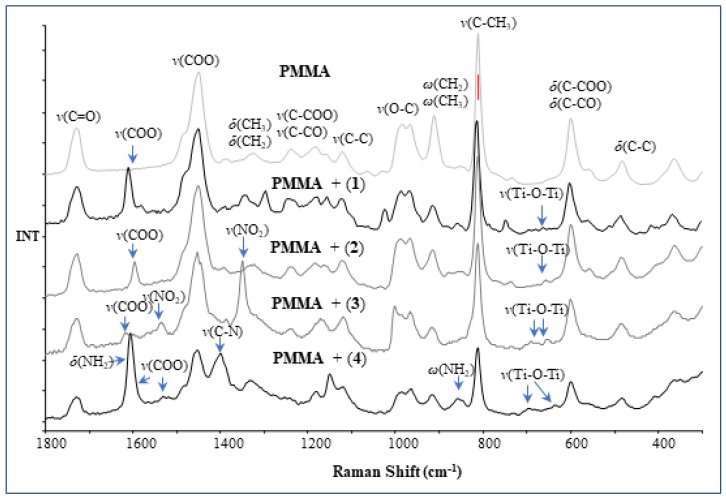
Raman spectra of poly(methyl methacrylate) (PMMA) and PMMA–titanium oxo-clusters (TOCs) composites. Arrows indicate the bands, derived from (1)–(4) complexes.

**Figure 5:**
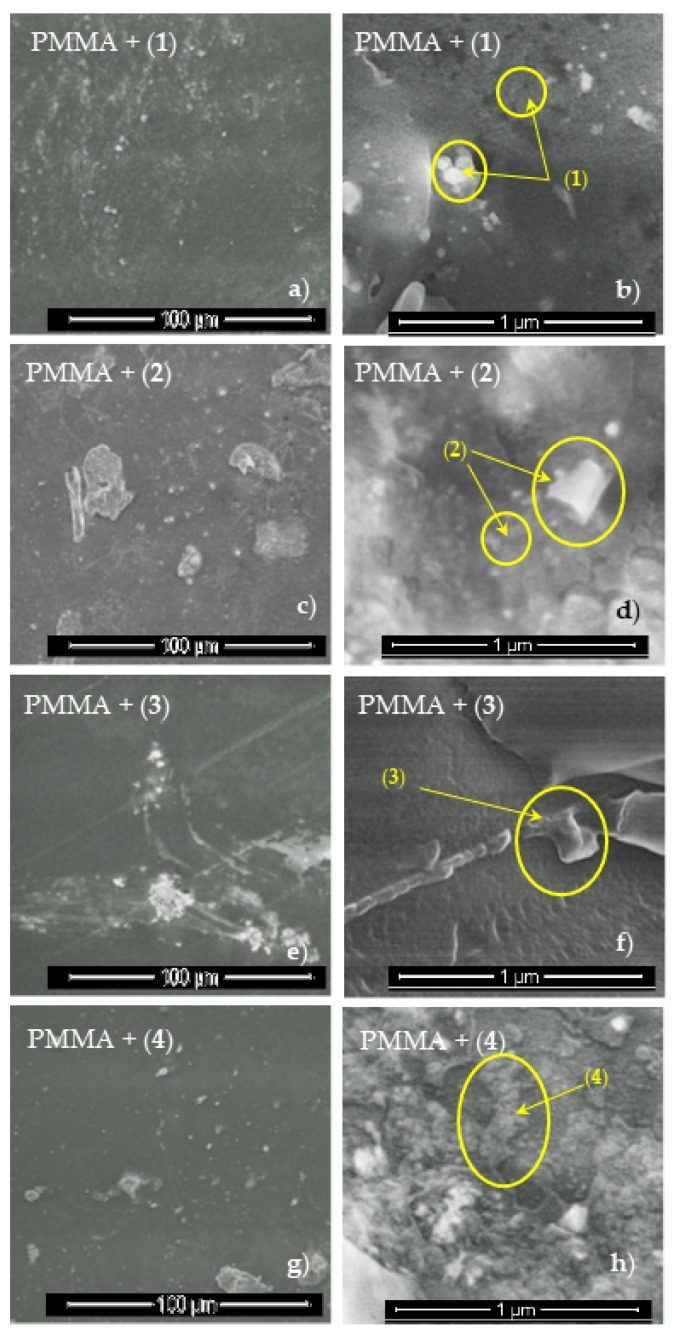
SEM images of PMMA + TOCs composites in different magnifications (**1**) (**a,b**), (**2**) (**c,d**), (**3**) (**e,f**), end (**4**) (**g,h**).

I In order to confirm the presence of the structurally stable Ti(IV) oxo-complexes in polymer, Raman spectra of composites were registered (Figure 4). Analysis of these data clearly indicates the presence of (**1**)–(**4**) complexes in the produced composite (PMMA + TOCs), which is evidenced by the appearance of bands attributed to the carboxylate ligands. Analysis of the region between 600 cm^−1^ and 750 cm^−1^, in which appears the bands coming from the modes of oxo-titanium bridges, is difficult due to overlapping of *ν*(Ti–O–Ti) bands and poly(methyl methacrylate) ones. The presence of micrograins and nanograins of complexes in polymer matrices were also seen in SEM images of PMMA + TOCs foils (Figure 5).

### 3.6. Photocatalytic Degradation of Methylene Blue (MB)

**Figure 6:**
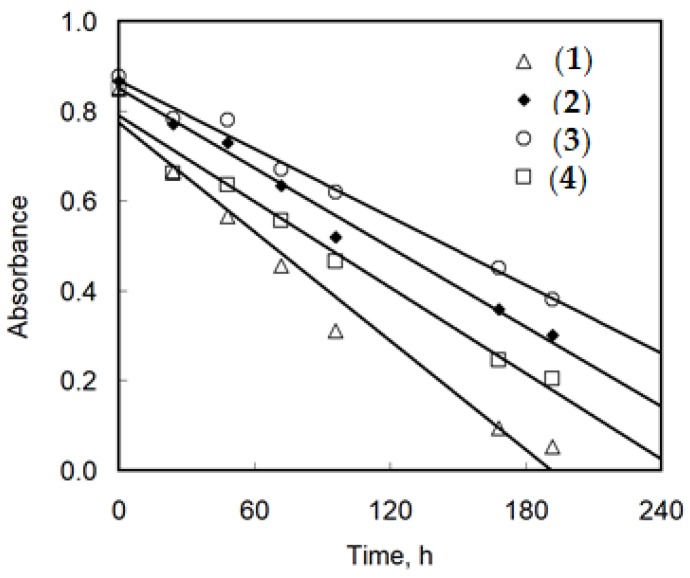
Changes in absorbance of methylene blue (MB) solution with time after addition of PMMA foil with a selected complex. MB = 1 × 10^−5^ M, PMMA foil surface = 0.64 cm^2^, *T* = r.t., *l* = 1 cm. Presented values are after blind test correction (R^2^ = 0.9701 (**1**), 0.9876 (**2**), 0.9896 (**3**), 0.9769 (**4**)).

To evaluate the influence of the different functionalities on photoinduced behavior, the photocalatytic degradation of MB were executed. The degradation of MB on polymer composites was followed by UV-Vis spectrophotometry by monitoring changes in the absorbance peak at 660 nm. Kinetic tests involved behavior of the studied complexes as well as blind tests (unmodified PMMA foil and sole MB solution). The changes in absorbance were roughly linear (Figure 6) which can be resulted the relatively small PMMA foil surfaces.

Thus, the rate of MB degradation was constant as long as the MB concentration was relatively high. Such behavior meant the negative values of slopes of the *A* = *f*(*t*) dependencies could be treated as the rate constants of MB photodegradation (Table 6).

Table 6 shows that the rate of MB degradation in darkness in case of sole MB and pure PMMA was very low and were similar. Under UV light, MB degraded more than 4 times faster. The PMMA foil accelerated its degradation by an additional 1.5 times (from 1.52 × 10^−3^ h^−1^ to 2.52 × 10^−3^ h^−1^). A correct comparison of the rate constants for PMMA foil with addition of titanium complexes should be referenced to the rate constant for pure PMMA foil “in darkness” (the last column in Table 6). The values of the corrected rate constants, presented in Table 6, indicated a trace photocatalytic activity of (**3**) in the MB degradation process. Complexes (**2**) and (**4**) were slightly more active and the para derivative was the best among those three complexes. The complex with fluorene (**1**) was clearly the most active in studied conditions.

### 3.7. ESR Evidence of Paramagnetic Species

**Figure 7:**
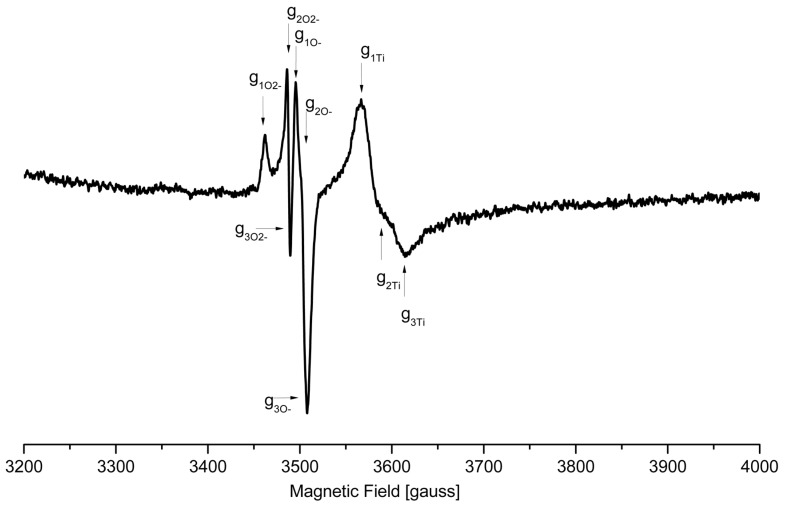
EPR spectrum of UV-Vis irradiated PMMA foil with complex (**1**).

To investigate the mechanism of photocatalytic process, the ESR analysis of powdered compounds and prepared materials was performed. Three types of paramagnetic species were observed for studied compounds (Table 7). Spectra of sample (**1**) (powder and PMMA foil) exhibited very weak radical signal (g_exp_ = 2.0031 and 2.0036, respectively) before UV-Vis irradiation. Due to unresolved g-tensor components, it was difficult to state with certainty what the structure of this radical was, but the value of the parameter indicated that it was O_2_^−^ rather than O^−^ [43]. Surprisingly, compound (**4**), before irradiation, exhibited signals of both types of oxygen radical (in case of powdered sample) and strong signal of O^−^ in case of the PMMA foil. For the other two samples, paramagnetic species before irradiation were not found. The most interesting ESR spectrum was observed for irradiated foil with sample (**1**) (with fluorene) (Figure 7). Spectrum—represented by signals originated in Ti(III) (centered in a distorted octahedral coordination field [44]), O_2_^−^, and O^−^ parameters—are given in Table 7. Similar spectrum was observed for the same but powdered sample, although the signals were much weaker, and Ti(III) signal was observed only as a trace. Irradiated sample (**2**) (foil and powder) exhibited spectra for which simulated g-parameter tensor components are typical of orthorhombic superoxide diatomic oxygen O_2_^−^ adsorbed on metal oxide surfaces [45]. However, the spectrum of foil was much better resolved than the spectrum of powder; signals observed for both kinds of samples and were characterized by the lowest intensity within the investigated samples. The signal is the result of electron trapping on Ti(IV) surface [46]:

e*^−^*+ Ti^4+^OH *−→* Ti^3+^OH (2)

Ti^3+^OH + O_2_*−→* Ti^4+^OH + *•*O_2_^−^ (3)

The main paramagnetic species observed on the spectra of samples (**3**) and (**4**) was V-type hole center [47]. Creation of this paramagnetic O^−^ species could be the result of the electron removal and radical being stabilized by reduction of Ti(IV) to Ti(III) [40]; the signal of Ti(III) might have been not observed due its broadness and nonintense line.

## 4. Discussion

The reaction of Ti(O^i^Bu)_4_ with fluorene-9-carboxylate acid, 3-chlorobenzoic acid, and 3-nitrobenzoic acid in molar ratio 4:1 at room temperature led to the formation of (Ti_4_O_2_(O^i^Bu)_10_(O_2_CR’)_2_) (R’ = C_13_H_9_ (**1**), C_6_H_4_Cl (**2**), C_6_H_4_NO_2_ (**3**)) oxo-clusters. Structural characterization of these compounds (single crystals X-ray diffraction and vibrational spectroscopy) and their comparison with the earlier studied (Ti_4_O_2_(O^i^Bu)_10_(O_2_CC_6_H_4_NH_2_)_2_) complex (**4**) [27] did not show a significant impact of the carboxylate ligand type on the {Ti_4_(µ_4_–O)(µ–O)} core structure. However, the clear {Ti_2_(µ_2_–O)} angle changes (which could be responsible for the facilitation of the photocatalytic process [26]) were observed, i.e., (**4**) < (**1**) < (**2**) < (**3**) (Table 2). The substantial difference in band structures based on used ligands revealed the results of DFT calculations. The presence of such carboxylic ligands as –O_2_CC_13_H_9_, –O_2_CC_6_H_4_Cl, and –O_2_CC_6_H_4_NH_2_ in structures of (**1**), (**2**), and (**4**), respectively, resulted in the *n*-doped band structure, while –O_2_CC_6_H_4_NO_2_ (**3**) was *p*-doped. The comparison of photoluminescence spectra of pure carboxylic ligands and synthesized compounds indicated the distinctive interactions between central oxo-titanium core and external ligands. Those interactions influenced the band gap value of produced materials, i.e., 3.59 eV for (**2**), 2.98 eV (**3**), 2.57 eV (**4**), and 2.55 eV (**1**) (the band gap of the widely used TiO_2_ anatase is 3.20 eV [22]). The obtained results were comparable with the previously studied Ti(IV) oxo-clusters containing functionalized carboxylate ligands [20]. The low value of the band gap of (**1**) can be explained by the presence of fluorene groups, whose derivatives are known for their photoluminescence properties [48].

Isolated crystals of all synthesized Ti(IV) oxo-complexes exhibited hydrophobic properties and very low sensitivity to the moisture. Therefore, in order to estimate the photocatalytic activity of the obtained compounds in the MB degradation process (UV irradiated), the composites produced by the introduction of (**1**)–(**4**) crystalline powders into the polymer matrix were used. The choice of PMMA as a polymer matrix was made on the basis of the spectroscopic criterion, i.e., the lack of PMMA bands in the Raman spectrum ranges in which the characteristic bands of oxo-complexes appear. This enabled the confirmation of the oxo-complex presence in the polymer matrix and the evaluation of potential interactions between the complex particles and the polymer matrix. Analysis of SEM images showed that PMMA + (**3**) foil contained dispersed large grains of complex (**3**) (Figure 5f), which was confirmed by the Raman spectrum of this composite (low intense bands at 1614 cm^−1^, 1659 cm^−1^ (ν(COO)), 1534 cm^−1^ (ν(NO_2_), 1008 cm^−1^ (ν(C–O), and 697,653 cm^−1^ (ν(Ti–O–Ti), (Figure 4, Table 3). Simultaneously, the lack of significant differences in the position and the shape of PMMA bands in PMMA + (**3**) spectrum suggested that significant interactions between polymer matrix and dispersed grains of (**3**) were not formed (Figure 4). The MB photodegradation studies (in UV) exhibited the trace activity of the PMMA + (**3**) composite, which could be associated with the *p*-doped band structure of (**3**) (other synthesized oxo-complexes are *n*-doped), the high value of band gap (2.98 eV). It is probable that the dispersion of large grains (micrograins/microcrystals) of the (**3**) complex in the PMMA matrix also contributed to the poor activity of this composite. The results obtained for (**3**) were in good agreement with earlier investigations of Liu et al. on the 3-nitrocarboxylate substituted compound (Ti_6_O_4_(O^i^Pr)_10_(O_3_P-Phen)_2_(O_2_CC_6_H_4_NO_2_)_2_) that also did not show any activity towards photocatalytic hydrogen production [23]. A better photocatalytic activity was observed for the PMMA + (**2**) foil (Figure 6 and Table 6). In addition, the spectral data analysis in that case did not indicate the formation interactions between (**2**) and PMMA (Figure 4, Table 3). Considering earlier reports [21], it should be noted that the presence of –O_2_CC_6_H_4_Cl ligands in the structure of (**2**) resulted in an increase in the value of the band gap up to 3.59 eV, which might have contributed to the lower photocatalytic activity of this material. Analysis of SEM images of PMMA + (**2**) showed that nanoparticles of (**2**)—whose diameter (*d*) is lower than 25 nm—and grains of *d* = 50 nm–300 µm formed a filling of a polymer matrix (Figure 5c,d). The substitution of the {Ti_4_O_2_} core by –O_2_CC_6_H_4_NH_2_ ligands promoted the decrease of band gap value (2.57 eV [20,26]) which was noted for the complex (**4**). Simultaneously, the SEM studies of PMMA + (**4**) foil proved that the filling of the polymer matrix was formed by islands composed of nanoparticles of *d* = 30–60 nm (Figure 5h). Distinct differences in the intensity and width of bands in Raman spectra of both oxo-cluster (**4**) and PMMA (Figure 4) suggested the formation of hydrogen bonds between the particles of the complex and the polymer (the hydrogen bonds between –NH_2_ groups of oxo-clusters and –C=O groups of the polymer are possible). In this case, low value of the band gap and possible weak interactions between amine groups of the oxo-cluster and PMMA had a direct influence on the increase of the photocatalytic activity of complex (**4**). A noticeable high photocatalytic activity was observed for foils produced by introduction of complex (**1**) into the PMMA matrix. Probable substitution of {Ti_4_O_2_} cores by fluorene-9-carboxylate ligands impacted the decrease of the band gap value (2.88 eV). Analysis of SEM images revealed that the densely packed nanoparticles of diameters *d =* 20–40 nm were the main filling of the PMMA + (**1**) foil (Figure 5b). The bands that were found in the Raman spectrum of PMMA + (1) at 1611 (m), 1581 (w) cm^−1^, 1023 (w) cm^−1^, and 660–700 (vw) cm^−1^ confirmed the presence of (**1**) in the polymer matrix (Figure 4, Table 3). Moreover, the comparison of PMMA + (**1**) and PMMA Raman spectra indicated lack of interactions between complex particles and the polymer matrix. It is interesting that ESR studies revealed the appearance of Ti(III) states during its irradiation from only the PMMA + (**1**) foil among the produced composite materials. According to works of Snoeberger et al. [49] and Negre et al. [50], the type of doping influences the mechanism of photoinduced charge transfer, promoting either electron or hole injection into functionalized oxo-titanium cluster [49,50]. Considering the result of the ESR and DFT studies, we can conclude that *n*-doping of POTs (in the case of (**3**)) did not allow for the photocatalytic activity due to absence of photogenerated Ti(III) states upon irradiation, while *p*-doping made it possible to generate Ti(III) and induce the photocatalytic action. DFT results revealed that HOMO orbitals of (**1**), (**2**), and (**4**) were located on ligands and LUMO on Ti atoms of the oxo-titanium core. Thus, upon irradiation, the charge was transferred to the core, generating Ti(III) states. This was confirmed by ESR experiment, which revealed Ti(III) states in PMMA–(**1**) composite foiled upon irradiation. According to the theoretical approach, these states should be present in (**2**) and (**4**), but the experiment did not confirm it. Possible explanation involves slower recombination of charges in compound with fluorene (**1**) than in ones with benzoic acid derivatives (**2**) and (**4**), which allows the detection of Ti(III).

## 5. Summary

The conducted works have led to an isolate and structural characterization of three new tetranuclear Ti(IV) oxo-clusters (TOCs) of the general formula (Ti_4_O_2_(O^i^Bu)_10_(OOCR’)_2_) from the 4:1 reaction mixture of Ti(O^i^Bu)_4_ with organic acids such as fluorene-9-carboxylate acid (**1**), 3-chlorobenzoic acid (2), and 3-nitrobenzoic acid (**3**). Analysis of structural and spectroscopic data exhibited that substitution of the {Ti_4_O_2_} core by the different carboxylate ligands did not significantly affect its architecture (the clear changes were found only for the value of Ti–(µ–O)–Ti angles). However, the use of different carboxylate ligands allowed control of the band gap value of produced oxo-clusters in the range of 3.6–2.6 eV. The photocatalytic activity of (**1**)–(**4**) was estimated on the basis of MB photodegradation processes (UV irradiated) that proceeded on the surface of a composite foils produced by the introduction of TOCs (20 wt %) into the PMMA matrix (PMMA + TOCs). The trace photocatalytic activity was noticed for (**3**), i.e., the *p*-doped (Ti_4_O_2_(O^i^Bu)_10_(O_2_CC_6_H_4_NO_2_)_2_) complex ((**1**), (**2**), (**4**) were *n*-doped). The highest photocatalytic activity was evidenced for PMMA + (**1**) foil, which contained (Ti_4_O_2_(O^i^Bu)_10_(O_2_CC_13_H_9_)_2_) (**1**) complex. According to results of our works, the following factors influence photoactivity of (**3**): *n*-dope character of the compound, relatively low band gap (2.55 eV), the presence of Ti(III) states upon irradiation evidenced by ESR spectrometry, and equal nanocrystals distribution in the PMMA matrix (confirmed by SEM imaging).

## Figures and Tables

**Table 1:** The selected crystal data and structure refinements for (Ti_4_O_2_(O^i^Bu)_10_(O_2_CR’)_2_) (R’ = C_13_H_9_ (**1**), PhCl (**2)**, PhNO_2_ (**3**); the complete crystallographic data are given in Table S1).

Parameters	(1)	(2)	(3)
Empirical formula	C_68_H_108_O_16_Ti_4_	C_54_H_98_Cl_2_O_16_Ti_4_	C_54_H_98_N_2_O_20_Ti_4_
Formula weight (g/mol)	1373.14	1265.82	1286.94
Temperature (K)	100(2)	100(2)	293(2)
Wavelength (Å)	0.89429	0.89429	0.71073
Space group	Monoclinic, P 1 21 1	Tetragonal, P 4 1	Triclinic, P 1
Unit cell dimensions (Å) and angles (°)	a = 12.447(3) b = 21.693(4) c = 14.071(3) α = γ = 90 β = 105.19(3)	a = 17.827(3) c = 41.475(8) α = β = γ = 90	a = 17.9713(9) b = 18.0686(7) c = 22.7006(9) α = 105.742(4) β = 98.362(4) γ = 92.631(4)
Volume (Å^3^)	3666.61(140)	13180.83(500)	6991.1(5)
Z, Calculated density (Mg/m^3^)	2, 1.244	8, 1.276	4, 1.223
Final R indices (I > 2sigma(I))	R_1_^a^ = 0.0444, wR_2_^b^ = 0.1182	R_1_^a^ = 0.0680, wR_2_^b^ = 0.1851	R_1_^a^ = 0.0860, wR_2_^b^ = 0.2304
Absolute structure parameter	0.041(3)	0.387(19)	N/A

^a^ R_1_ = Σ||*F_0_*| − |*F_c_*||/Σ|*F_0_*|; ^b^ wR_2_ = (Σw(*F_0_*^2^ − *F_c_*^2^)^2^/Σ(w(*F_0_*^2^)^2^) )^1/2^. CCDC 1826273, 1826276, and 1826277 contain the supplementary crystallographic data for (**1**), (**2**), and (**3**), respectively. These data can be obtained free of charge via http://www.ccdc.cam.ac.uk/conts/retrieving.html or from the Cambridge Crystallographic Data Centre, 12 Union Road, Cambridge CB2 1EZ, UK; fax: (+44) 1223-336-033; or e-mail: deposit@ccdc.cam.ac.uk.

**Table 2:** Selected bond lengths (Å) and angles (°) of (Ti_4_O_2_(O^i^Bu)_10_(O_2_CR’)_2_) (R’ = C_13_H_9_ (**1**), PhCl (**2**), PhNO_2_ (**3**)).

Parameter		(1)	(2)	(3)
Distances (Å)				
Ti–Ti	Ti1–Ti3	3.1690(11)	3.2016(16)	3.2060(12)
	Ti1–Ti2	2.9427(12)	2.9488(16)	2.9521(13)
	Ti1–Ti4	3.1446(12)	3.1456(15)	3.1508(12)
	Ti2–Ti3	3.1566(11)	3.1532(17)	3.1617(13)
	Ti2–Ti4	3.1738(13)	2.1859(17)	3.2009(12)
	Ti3–Ti4	4.0386(16)	3.9255(16)	3.9510(13)
Ti–(µ_4_–O)	Ti1–O2	2.0457(30)	2.0599(39)	2.059(3)
	Ti2–O2	2.0357(28)	2.0622(41)	2.052(3)
	Ti3–O2	2.0820(27)	2.0603(38)	2.074(3)
	Ti4–O2	2.0896(27)	2.0264(38)	2.042(3)
Ti–(µ_2_–O)	Ti1–O3	1.8326(37)	1.8643(40)	1.839(3)
	Ti3–O3	1.8499(41)	1.8182(39)	1.826(3)
Ti–(µ_2_–OR)	Ti1–O11	1.9609(27)	1.9515(37)	1.944(3)
	Ti1–O1	2.0042(28)	2.0133(38)	2.017(3)
	Ti2–O21	1.9979(27)	2.0095(38)	2.020(3)
	Ti2–O31	1.9633(28)	1.9858(38)	1.969(3)
	Ti3–O1	1.9896(36)	2.0041(39)	1.993(3)
	Ti3–O31	2.0981(29)	2.0698(45)	2.089(3)
	Ti4–O11	2.0944(32)	2.1355(43)	2.114(3)
	Ti4–O21	1.9985(30)	2.0182(44)	2.004(3)
Ti–O (carb)	Ti4–O111	2.027(3)	2.051(4)	2.068(4)
	Ti1–O112	2.195(3)	2.135(4)	2.145(3)
	Ti2–O131	2.190(3)	2.127(5)	2.142(4)
	Ti3–O132	2.028(3)	2.073(5)	2.056(4)
O–C (carb)	O111–C112	1.270(5)	1.272(8)	1.267(6)
	O112–C112	1.245(5)	1.244(8)	1.240(6)
	O131–C132	1.245(6)	1.259(9)	1.260(7)
	O132–C132	1.257(6)	1.255(9)	1.245(6)
Angles (°)				
Ti–(µ_4_–O)–Ti	Ti3–O2–Ti2	100.10(12)	99.80(17)	100.05(12)
	Ti3–O2–Ti1	100.31(13)	102.02(17)	101.76(13)
	Ti2–O2–Ti1	92.28(12)	91.36(17)	91.79(12)
	Ti3–O2–Ti4	150.98(16)	147.7(2)	147.43(15)
	Ti2–O2–Ti4	100.58(13)	102.34(18)	102.83(14)
	Ti1–O2–Ti4	98.99(12)	100.66(18)	100.39(12)
Ti–(µ_2_–O)–Ti	Ti1–O3–Ti2	106.09(15)	106.5(2)	107.36(16)
Ti–(µ_2_–OR)–Ti	Ti1–O1–Ti3	105.04(15)	105.72(18)	106.16(14)
	Ti1–O11–Ti4	101.65(13)	100.52(19)	101.79(13)
	Ti2–O31–Ti3	101.97(14)	102.04(18)	102.35(13)
	Ti2–O21–Ti4	105.16(14)	104.55(19)	105.38(15)
O–C–O (carb)	O111–C112–O112	126.0(4)	125.5(6)	125.5(5)
	O131–C132–O132	126.6(4)	126.1(6)	125.8(5)

**Table 3:** Results of vibrational spectra studies of (**1**)–(**3**) complexes.

Modes	(1)		(2)		(3)	
	IR	R	IR	R	IR	R
ν_as_(COO)	1566 (s)	1611 (s) 1581 (w)	1590 (s) 1551 (m)	1595 (s) 1552 (m)	1595 (m) 1559 (s)	1614 (w) 1599 (vw) 1561 (m)
ν_s_(COO)	1448 (m)	1450 (m)	1442 (m)	1459 (m)	1434 (m)	1456 (s)
ν_as_(NO_2_)	-	-	-	-	1530 (m)	1534 (m)
ν_s_(NO_2_)	-	-	-	-	1347 (s)	1349 (m)
ν_a_(Ti–µ–O–Ti) ν_s_(Ti–µ–O–Ti)	712 (w)	700 (m) 666 (m)	-	699 (m) 661 (m)	-	697 (m) 653 (m)
ν_a_(Ti–µ_4_–O–Ti) ν_s_(Ti–µ_4_–O–Ti)	636 (m) 546 (s)	543 (vw) 414 (m)	635 (m) 547 (m)	548 (vw) 415 (m)	643 (m) 546 (s)	536 (vw) 412 (m)

**Table 4:** The calculated frequencies of the (Ti–O–Ti) modes of {Ti_2_-(µ_2_-O)} and {Ti_4_-(µ_4_-O)} bridges. In DFT calculations of (**1**)–(**3**) complexes the O^i^Bu ligands were exchanged on the OMe one.

Complex	Mode	Frequency (cm^−1^)
(Ti_4_O_2_(OMe)_10_(O_2_CC_13_H_9_)_2_) (**1**)	ν_a_(Ti–µ–O–Ti) ν_s_(Ti–µ–O–Ti) ν_a_(Ti–µ_4_–O–Ti) ν_s_(Ti–µ_4_–O–Ti)	703 700 623 570,439
(Ti_4_O_2_(OMe)_10_(O_2_CC_6_H_4_Cl)_2_) (**2**)	ν_a_(Ti–µ–O–Ti) ν_s_(Ti–µ–O–Ti) ν_a_(Ti–µ_4_–O–Ti) ν_s_(Ti–µ_4_–O–Ti)	705 700 622 560,448
(Ti_4_O_2_(OMe)_10_(O_2_CC_6_H_4_NO_2_)_2_) (**3**)	ν_a_(Ti–µ–O–Ti) ν_s_(Ti–µ–O–Ti) ν_a_(Ti–µ_4_–O–Ti) ν_s_(Ti–µ_4_–O–Ti)	706 699 622 567,444

**Table 5:** Comparison of experimentally determined band gap values (using diffuse reflectance spectra), and theoretically calculated (HSE06/6-31(d,p) level of theory in calculations of (**1**)–(**3**) complexes the O^i^Bu ligands were exchanged on the OMe one).

Complex	Calculated Band Gap (eV)	Experimental Band Gap (eV)
(Ti_4_O_2_(OMe)_10_(O_2_CC_13_H_9_)_2_) (**1**)	3.93	2.55
(Ti_4_O_2_(OMe)_10_(O_2_CC_6_H_4_Cl)_2_) (**2**)	4.73	3.59
(Ti_4_O_2_(OMe)_10_(O_2_CC_6_H_4_NO_2_)_2_) (**3**)	4.30	2.98

**Table 6:** Rate constants of MB degradation for sole MB, pure PMMA, and PMMA with addition of the studied materials.

Sample	10^3^ Rate Constant, h^−1^	Sample	10^3^ Rate Constant, h^−1^	10^3^ Rate Constant in Reference to PMMA, h^−1^
sole MB in darkness	0.35 ± 0.15	(**1**) in light	4.03 ± 0.32	1.51 ± 0.36
sole MB in light	1.52 ± 0.09	(**2**) in light	2.95 ± 0.15	0.43 ± 0.22
PMMA in darkness	0.26 ± 0.04	(**3**) in light	2.53 ± 0.12	0.01 ± 0.20
PMMA in light	2.52 ± 0.16	(**4**) in light	3.19 ± 0.22	0.67 ± 0.27

**Table 7:** Results of EPR studies for UV-Vis irradiated complexes (PMMA foils) ((Ti_4_O_2_(O^i^Bu)_10_(O_2_CR’)_2_) (R’ = C_13_H_9_ (**1**), PhCl (2), PhNO_2_ (**3**), PhNH_2_ (**4**)).

Sample	g Parameters			Species
PMMA +	g_1_	g_2_	g_3_	
(**1**)	2.024	2.0095	2.0034	O_2_^−^
	1.967	1.957	1.938	Ti(III)
	2.003	-	1.997	O^−^
(**2**)	2.024	2.0095	2.0034	O_2_^−^
(**3**)	2.0185	2.0052	1.987	O^−^
(**4**)	2.0182	2.005	1.987	O^−^

## Supplementary Materials

The following are available online at http://www.mdpi.com/1996-1944/11/9/1661/s1, Figure S1: Infrared and Raman spectra of (**1**)–(**3**) complexes, Figure S2: The calculated partial density of states of oxo-complexes (**1**), (**2**), and (**3**), Figure S3: DFT calculated HOMO (top) and LUMO (bottom) molecular orbitals of (**1**), (**2**) and (**3**), **Table S1:** Comparison of the experimental and the calculated (DFT) structural data of {Ti_4_O_2_} cores of studied oxo-clusters: [Ti_4_O_2_(O^i^Bu)_10_(O_2_CC_13_H_9_)_2_] (**1**), [Ti_4_O_2_(O^i^Bu)_10_(O_2_CC_6_H_4_Cl)_2_] (**2**), [Ti_4_O_2_(O^i^Bu)_10_(O_2_CC_6_H_4_NO_2_)_2_] (**3**), [Ti_4_O_2_(OMe)_10_(O_2_CC_13_H_9_)_2_] DFT(**1**), [Ti_4_O_2_(OMe)_10_(O_2_CC_6_H_4_Cl)_2_] DFT(**2**), [Ti_4_O_2_(OMe)_10_(O_2_CC_6_H_4_NO_2_)_2_] DFT (**3**).
