# Supplementary material for: Tetranuclear Oxo-Titanium Clusters with Different Carboxylate Aromatic Ligands: Optical Properties, DFT Calculations, and Photoactivity
Source: Materials (Basel). 2018 Sep 8;11(9):1661. doi: 10.3390/ma11091661 (PMC6164694; doi:10.3390/ma11091661)
Supplement: Supplementary file 1 [file materials-11-01661-s001.pdf]

materials

Supporting information

Tetranuclear oxo-titanium clusters with different aromatic ligands: optical properties, DFT calculations, and photoactivity

Maciej Janek [1], Tadeusz M. Muzioł [1], Aleksandra Radtke [1,2], Maria Jerzykiewicz [2], Piotr Piszczek[1,2,*]

[1] Faculty of Chemistry, Nicolaus Copernicus University in Toruń, ul. Gagarina 7, 87-100 Toruń, Poland; maciejjanekk@gmail.com (M.Jk.); Aleksandra.Radtke@umk.pl (A.R.); tadeuszmuziol@wp.pl (T.M.M.)
[2] Nano-implant Ltd. Gagarina 5/102, 87-100 Toruń, Poland, NIP 9562314777;
[3] Faculty of Chemistry, Wrocław University, ul. F. Joliot-Curie 14, 50-383 Wrocław, Poland; maria.jerzykiewicz@chem.uni.wroc.pl
[*] Correspondence: piszczek@chem.umk.pl; Tel.: +48-56-611-45-92

Table S1. Comparison of the experimental and the calculated (DFT) structural data of {Ti_4O_2} cores of studied oxo-clusters: [$Ti_4O_2(O^iBu)_{10}(O_2CC_{13}H_9)_2$] (**1**), [$Ti_4O_2(O^iBu)_{10}(O_2CC_6H_4Cl)_2$] (**2**), [$Ti_4O_2(O^iBu)_{10}(O_2CC_6H_4NO_2)_2$] (**3**),. [$Ti_4O_2(OMe)_{10}(O_2CC_{13}H_9)_2$] DFT(**1**), [$Ti_4O_2(OMe)_{10}(O_2CC_6H_4Cl)_2$] DFT(**2**), [$Ti_4O_2(OMe)_{10}(O_2CC_6H_4NO_2)_2$] DFT (**3**).

		(1)	DFT (1)	(2)	DFT (2)	(3)	DFT (3)
				Distances [Å]			
Ti-Ti	Ti1-Ti3	3.1690(11)	3.1875	3.2016(16)	3.1849	3.2060(12)	3.1855
	Ti1-Ti2	2.9427(12)	2.9149	2.9488(16)	2.9132	2.9521(13)	2.9121
	Ti1-Ti4	3.1446(12)	3.1480	3.1456(15)	3.1439	3.1508(12)	3.1461
	Ti2-Ti3	3.1566(11)	3.1478	3.1532(17)	3.1439	3.1617(13)	3.1461
	Ti2-Ti4	3.1738(13)	3.1877	2.1859(17)	3.1849	3.2009(12)	3.1855
	Ti3-Ti4	4.0386(16)	4.0173	3.9255(16)	4.0270	3.9510(13)	4.0246
Ti-(μ4-O)	Ti1-O2	2.0457(30)	2.0504	2.0599(39)	2.0472	2.059(3)	2.0466
	Ti2-O2	2.0357(28)	2.0504	2.0622(41)	2.0472	2.052(3)	2.0466
	Ti3-O2	2.0820(27)	2.0764	2.0603(38)	2.0797	2.074(3)	2.0796
	Ti4-O2	2.0896(27)	2.0767	2.0264(38)	2.0797	2.042(3)	2.0796
Ti-(μ2-O)	Ti1-O3	1.8326(37)	1.8177	1.8643(40)	1.8184	1.839(3)	1.8178
	Ti3-O3	1.8499(41)	1.8178	1.8182(39)	1.8184	1.826(3)	1.8178
Ti-(μ2-OR)	Ti1-O11	1.9609(27)	1.9600	1.9515(37)	1.9581	1.944(3)	1.9563
	Ti1-O1	2.0042(28)	2.0066	2.0133(38)	2.0138	2.017(3)	2.0147
	Ti2-O21	1.9979(27)	2.0067	2.0095(38)	2.0138	2.020(3)	2.0147
	Ti2-O31	1.9633(28)	1.9599	1.9858(38)	1.9581	1.969(3)	1.9564
	Ti3-O1	1.9896(36)	1.9988	2.0041(39)	1.9938	1.993(3)	1.9911
	Ti3-O31	2.0981(29)	2.0897	2.0698(45)	2.0826	2.089(3)	2.0850
	Ti4-O11	2.0944(32)	2.0897	2.1355(43)	2.0826	2.114(3)	2.0850
	Ti4-O21	1.9985(30)	1.9987	2.0182(44)	1.9938	2.004(3)	1.9911
				Angles [deg]			
Ti-(μ4-O)-Ti	Ti3-O2-Ti2	100.10(12)	99.41	99.80(17)	99.25	100.05(12)	99.36
	Ti3-O2-Ti1	100.31(13)	101.14	102.02(17)	101.02	101.76(13)	101.07
	Ti2-O2-Ti1	92.28(12)	90.60	91.36(17)	90.72	91.79(12)	90.704
	Ti3-O2-Ti4	150.98(16)	150.62	147.7(2)	151.01	147.43(15)	150.773
	Ti2-O2-Ti4	100.58(13)	101.14	102.34(18)	101.02	102.83(14)	101.07

	Ti1-O2-Ti4	98.99(12)	99.42	100.66(18)	99.25	100.39(12)	99.36
Ti-(μ2-O)-Ti	Ti1-O3-Ti2	106.09(15)	106.60	106.4(2)	106.46	107.32(15)	106.45
Ti-(μ2-OR)-Ti	Ti1-O1-Ti3	105.04(15)	105.46	105.72(18)	105.26	106.16(14)	105.35
	Ti1-O11-Ti4	101.65(13)	101.99	100.52(19)	102.12	101.79(13)	102.20
	Ti2-O31-Ti3	101.97(14)	101.98	102.04(18)	102.12	102.35(13)	102.19
	Ti2-O21-Ti4	105.16(14)	105.47	104.55(19)	105.26	105.38(15)	105.35

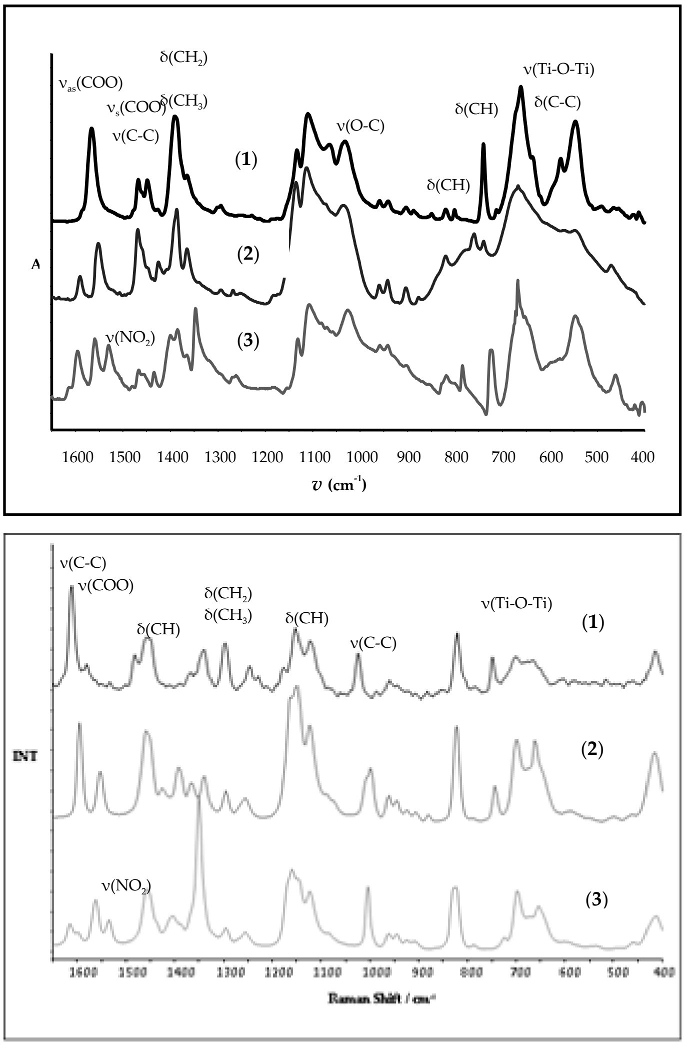

Figure S1. Infrared and Raman spectra of (**1**)–(**3**) complexes.

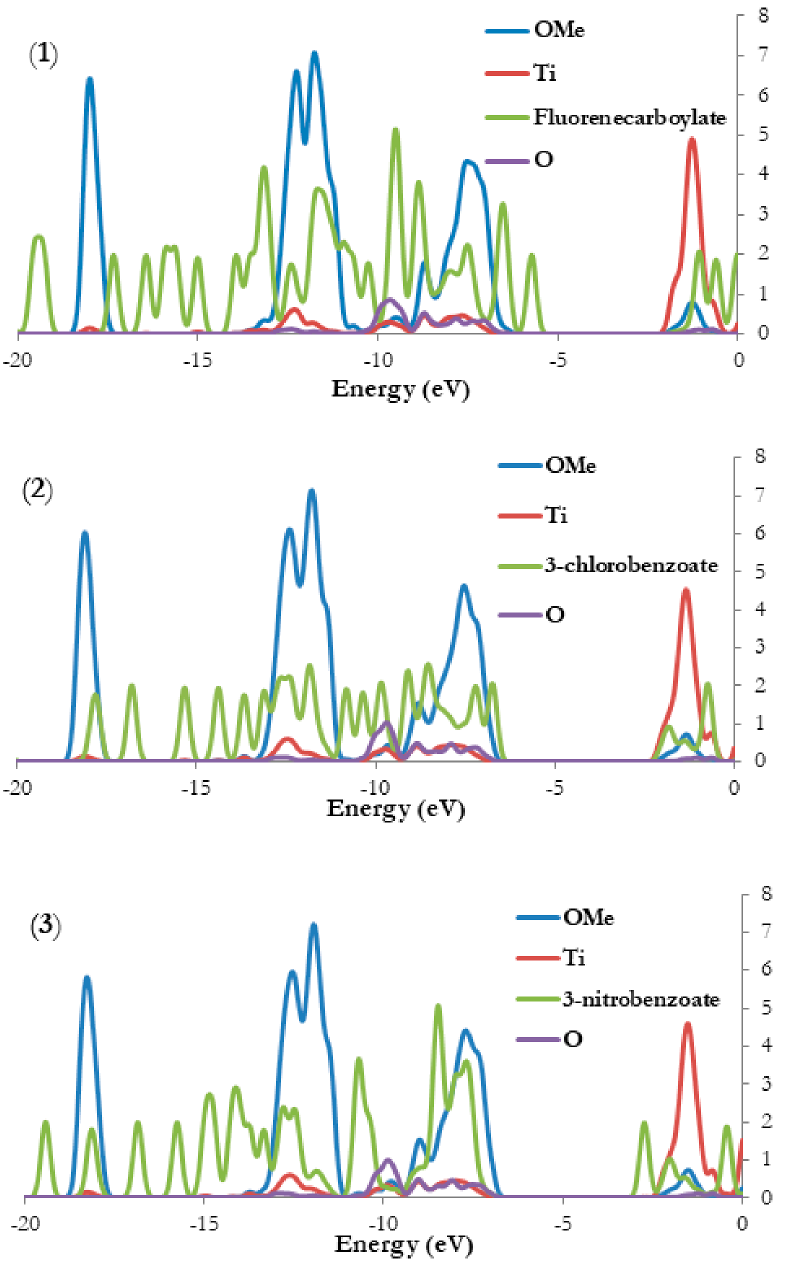

Figure S2. The **c**alculated partial density of states of oxo-complexes (**1**), (**2**), and (**3**).

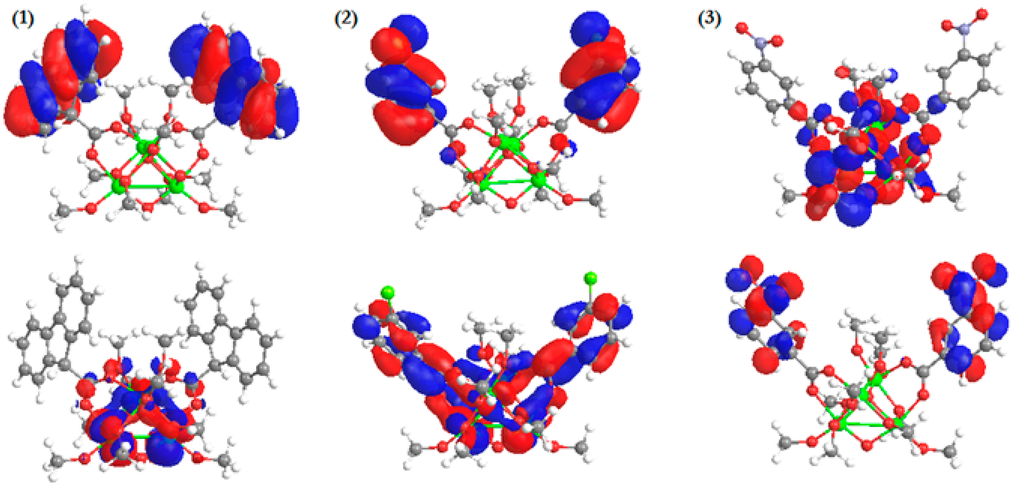

Figure S3. DFT calculated HOMO (top) and LUMO (bottom) molecular orbitals of (**1**), (**2**) and (**3**).
